# Two distinct groups of porcine enteropathogenic *Escherichia coli *strains of serogroup O45 are revealed by comparative genomic hybridization and virulence gene microarray

**DOI:** 10.1186/1471-2164-10-402

**Published:** 2009-08-26

**Authors:** Guillaume Bruant, Yongxiang Zhang, Philippe Garneau, Justin Wong, Chad Laing, John M Fairbrother, Victor PJ Gannon, Josée Harel

**Affiliations:** 1 Groupe de Recherche sur les Maladies Infectieuses du Porc, Faculté de médecine vétérinaire, Université de Montréal, 3200 rue Sicotte, Saint-Hyacinthe, Québec J2S 7C6, Canada; 2 Laboratory for Foodborne Zoonoses, Public Health Agency of Canada, Lethbridge, Alberta, T1J 3Z4, Canada

## Abstract

**Background:**

Porcine enteropathogenic *Escherichia coli *(PEPEC) strains of serogroup O45 cause post-weaning diarrhea and produce characteristic attaching and effacing (A/E) lesions. Most O45 PEPEC strains possess the locus of enterocyte effacement (LEE), encoding the virulence factors required for production of A/E lesions, and often possess the *paa *gene, which is thought to contribute to the early stages of PEPEC pathogenicity. In this study, nine O45 PEPEC strains and a rabbit enteropathogenic (REPEC) strain, known to produce A/E lesions *in vivo*, were characterized using an *E. coli *O157-*E. coli *K12 whole genome microarray and a virulence gene-specific microarray, and by PCR experiments.

**Results:**

Based on their virulence gene profiles, the 10 strains were considered to be atypical EPEC. The differences in their genomes pointed to the identification of two distinct evolutionary groups of O45 PEPEC, Groups I and II, and provided evidence for a contribution of these genetic differences to their virulence in pigs. Group I included the REPEC strain and four O45 PEPEC strains known to induce severe A/E lesions in challenged pigs whereas Group II was composed of the five other O45 PEPEC strains, which induced less severe or no A/E lesions in challenged pigs. Significant differences between Groups I and II were found with respect to the presence or absence of 50 O-Islands (OIs) or S-loops and 13 K-islands (KIs) or K-loops, including the virulence-associated islands OI#1 (S-loop#1), OI#47 (S-loop#71), OI#57 (S-loop#85), OI#71 (S-loop#108), OI#115, OI#122, and OI#154 (S-loop#253).

**Conclusion:**

We have genetically characterized a collection of O45 PEPEC strains and classified them into two distinct groups. The differences in their virulence gene and genomic island content may influence the pathogenicity of O45 PEPEC strains, and explain why Group I O45 PEPEC strains induced more severe A/E lesions in explants and challenged pigs than Group II strains.

## Background

*Escherichia coli *of serogroup O45 may be isolated both in intestinal and extraintestinal sites, although they have been only sporadically described in the latter [1-3]. On the other hand, intestinal *E. coli *strains have been more frequently identified as belonging to this serogroup. Intestinal O45 *E. coli *strains have been isolated from animals and humans and have been classified as both enterotoxigenic (ETEC) and attaching and effacing (AEEC) *E. coli*, the latter including both enterohemorrhagic (EHEC) and enteropathogenic (EPEC) *E. coli *[4-6]. Serogroup O45 is particularly important among porcine EPEC (PEPEC) strains which cause post-weaning diarrhea (PWD) characterized by specific attaching and effacing (A/E) lesions [7-9]). Most O45 PEPEC strains possess the locus of enterocyte effacement (LEE) pathogenicity island, which contains virulence genes necessary for the production of A/E lesions. They also often possess the *paa *gene (for porcine A/E associated gene), which encodes a virulence factor involved in the A/E phenotype and is thought to contribute to the early stages of PEPEC pathogenicity [10]. These strains also have the ability to produce A/E lesions in experimentally inoculated newborn gnotobiotic piglets and in a homologous *in vitro *model using newborn piglet ileal explants, as well as to adhere to PK15 porcine kidney cells *in vitro *[10-14].

Genomic islands (GIs) such as LEE are regions of bacterial genomes that have been acquired by horizontal gene transfer and often contain blocks of genes that function together in specific processes. When the genomes of the two *E. coli *O157:H7 strains EDL933 and Sakai were compared with that of *E. coli *K12 strain MG1655, the GIs found to be present in strains EDL933 and Sakai but absent in strain MG1655 were named O-islands (OIs) and Sakai loops (S-loops), respectively. The GIs found to be present in *E. coli *K12 but absent from the two *E. coli *O157:H7 strains were named K-islands (KIs) and K-loops, respectively [15-17]. GIs related to the virulence of a pathogen are also referred to as pathogenicity islands (PAIs) [18]. In *E. coli *O157:H7 strain EDL933, several large OIs encode virulence or putative virulence factors. These OIs include OI#45 (S-loop#69) and OI#93 (S-loop#153) for Shiga toxin 2 and 1, respectively, OI#148 (S-loop#244) for LEE, and OI#57 (S-loop#85) for *paa *[16,17].

A recent microarray-based study has catalogued genomic alterations in a collection of *E. coli *O157:H7 strains, particularly in GIs, suggesting the existence of two dominant lineages, with characteristics that are unique to each of them [19]. Previous studies performed on various AEEC strains have also shown that, depending on their pathotype and host specificity, strains can show variations in their LEE sequences as well as in the site of insertion of LEE in the chromosome [14,20,21]. The purpose of the present study was to examine the genotypic differences, particularly in LEE sequences and chromosomal insertion sites, and in the presence or absence of non LEE-encoded virulence factors, such as Paa, among a collection of O45 PEPEC strains which have been previously shown to induce A/E lesions in pigs. In this study, we have characterized O45 PEPEC strains using a DNA-microarray designed specifically for detection of *E. coli *virulence genes [22] and compared their genomes using comparative genomic hybridization (CGH) and PCR. We identified two distinct groups of PEPEC O45 strains, between which there were significant variations in GI content.

## Methods

### Bacterial strains and preparation of genomic DNA

Nine O45 PEPEC strains, which were isolated at the Faculté de médecine vétérinaire, Saint-Hyacinthe, Québec, Canada, from pigs with PWD [13] were used for the microarray studies (Table 1). These strains were selected based, i) on their ability to produce or not A/E lesions in challenged pigs [13] and in an homologous *ex vivo *model using newborn piglet ileal explants (data not shown), ii) and on the severity of the A/E manifestation they produced [13] (Table 1). Because of its genetic and phenotypic similarities with the O45 PEPEC strains [23], the O103 rabbit EPEC (REPEC) strain E22, provided by Eric Oswald (INRA, Toulouse, France) [24,25], was included in the study. Five *E. coli *reference strains were used as controls in PCR experiments: the two O157:H7 *E. coli *strains EDL933 and Sakai, the K12 strain MG1655, the uropathogenic (UPEC) strain CFT073 and the REPEC strain RDEC-1.

**Table 1:** Characteristics of O45 PEPEC strains and REPEC strain E22 used in this study.

Name^a^	Serogroup	Origin	A/E lesions in explants^b^	A/E lesions in challenged piglets^c^		
				
				Extent^d^	Site^e^	Inflammation^f^
E22^g^	O103:H2	Rabbit	ND	ND	ND	ND
ECL1001 (86–1390)	O45:KE65	Pig	+	++++	I/Ce/C-P/C-D	S
ECL2004 (81–4420)	O45:KE65	Pig	+	++++	I/Ce/C-P/C-D	L
ECL2017 (86–4220)	O45:KE65	Pig	+	+++	I/Ce/C-P/C-D	M
ECL2033 (91-19-172)	O45:KE65	Pig	+	++++	I/Ce/C-P/C-D	-
ECL2019 (88–1861)	O45:KE65	Pig	-	-	-	-
ECL2020 (88–4299)	O45:KE65	Pig	+	++	C-P/C-D	L
ECL2027 (89-56-196)	O45:KE65	Pig	-	-	-	-
ECL2076^h ^(87–4725)	O45:KE65	Pig	+			
ECL2078 (83–2315)	O45:KE65	Pig	+	++	D/I/Ce/C-P/C-D	L

^a ^Former names used in a previous study by Zhu *et al*. [13] are indicated in parentheses.

^b ^Unpublished data from studies performed in our laboratory. +, strain tested produced A/E lesions in explants; -, no lesion observed; ND, not determined.

^c ^Data from a previous study by Zhu *et al*. [13].

^d ^++++, extensive bacterial colonization and severe effacement of microvilli; +++, large areas of bacterial colonization and heavy effacement; ++, focal lesions; +, small scattered focal lesions; -, no lesions observed; ND, not determined.

^e ^A/E lesions were observed in duodenum (D), ileum (I), cecum (Ce), proximal colon (C-P), and distal colon (C-D); ND, not determined.

^f ^L, light inflammatory response; M, mild inflammatory response; S, severe inflammatory response; -, no inflammatory response; ND, not determined.

^g ^REPEC reference strain [24,25].

^h ^Strain ECL2076 produced A/E lesions in explants but was not included in the study by Zhu *et al*. [13].

For DNA preparations, strains were grown overnight in 45 mL of Brain-Heart-Infusion (BHI) broth at 37°C. The cultures were centrifuged at 8000 rpm for 10 minutes and the pellet was dissolved in 15 mL of 10 mM NaCl, 20 mM Tris-HCl (pH 8.0), 1 mM EDTA, 100 μg/mL proteinase K and 0.5% SDS. This suspension was incubated at 50°C for 2 h and DNA was extracted with an equal volume of phenol:chloroform:isoamyl alcohol (25:24:1). Following centrifugation for 10 min at 8000 rpm, the upper phase was removed and precipitated by adding 0.1 volume of 3 M NaOAc (pH 5.2) and 2 volumes of 99% ethanol. The DNA precipitate was then spooled out of the solution using a sterile glass rod, washed with 70% ethanol, and dissolved in 5 mL of TE (10 mM Tris-HCl, 1 mM EDTA, pH 8.0) buffer.

### *E. coli *DNA microarrays

For the whole genome microarray (named *E. coli *O157:H7 microarray), Corning Ultra-Gap II slides (Corning, Acton, MA) were spotted with the MWG *E. coli *O157:H7 array set (MWG Biotech). The MWG array set consists of 6167 50-mer oligonucleotides covering the whole genomes of *E. coli *K-12 strain MG1655 [15] and *E. coli *O157:H7 strains Sakai (RIMD 0509952) [16] and EDL933 (ATCC700927) [17].

The *E. coli *virulence microarray used in this study was derived from the one previously developed by Bruant *et al*. and included 315 70-mer oligonucleotides specific for 189 *E. coli *virulence or putative virulence genes or markers found in various intestinal and extraintestinal *E. coli *strains of all known pathotypes [22]. Probes were specific for genes encoding adhesins; toxins; bacteriocins; anti-aggregative factors; autotransporters; capsular, flagellar, and somatic antigens; hemolysins; invasins; iron acquisition systems or transport proteins; and outer membrane proteins, as well as other genes recently shown to be associated with virulence in *E. coli*. This microarray also detected genetic variants of particular genes, such as the intimin-encoding gene *eae *(variants alpha, alpha2, beta, beta2, delta, epsilon, epsilon2, eta, gamma, gamma2, iota, iota2, lambda, mu, nu, pi, xi, and zeta), *espA *(variants *espA1*, *espA2*, and *espA3*), *espB *(variants *espB1*, *espB2*, and *espB3*), and *tir *(variants *tir-1*, *tir-2*, and *tir-3*) from the LEE. Oligonucleotides specific for three variants of the major fimbrial subunit of the long polar fimbria (LPF)-encoding gene *lpfA *were also included. These were based on sequences from the *lpfA *genes of EPEC strains of serogroup O113 (*lpfA*_O113_), OI#141 from *E. coli *of serotype O157:H7 (*lpfA1*), and REPEC strains and *E. coli *of serogroup O26 (*lpfA*_R141_).

### Microarray hybridizations

Prior to *E. coli *O157:H7 microarray hybridization, each array was pre-hybridized at 50°C in a solution of 5 × SSC, 0.1% SDS and 0.1% BSA for 1 h. Following this step, arrays were washed completely in dH_2_O, rinsed with isopropanol, and then centrifuged and dried. For hybridization, 5 μg of test genomic DNA were digested with *Eco*RV and *Pst*I restriction enzymes, 3 μg of which were labeled with ULYSIS Alexa Fluor 647 dye (Invitrogen, Burlington, ON). Genomic DNA from strains MG1655, Sakai and EDL933 was digested in an analogous fashion, and 1 μg of the preparation from each strain was combined and labeled with Alexa Fluor 546 dye (Invitrogen, Burlington, ON). This labeled genomic DNA mixture was then used as a reference for all hybridizations. Unincorporated dye was removed using Qiaquick PCR purification kits (Qiagen, Mississauga, ON), according to the manufacturer's instructions, and DNA was eluted in 30 μl of 0.1 × TE buffer. Labeled DNA was vacuum-dried and resuspended in 20 μl of dH_2_O. A 70 μl hybridization solution consisting of 30% formamide, 5 × SSC, 0.1% SDS, 0.1 mg/ml sonicated Salmon sperm DNA, and equal amounts of test and reference labeled DNAs, each containing at least 30 pmol of incorporated dye, was denatured at 95°C for 5 min and briefly centrifuged to collect all the contents. DNA preparations were then hybridized overnight (16 h) at 42°C. After hybridization, arrays were washed according to the modified Corning method (Corning). Arrays were then scanned with a GenePix 4000B scanner (Axon Instruments, Redwood City, CA) and processed using GenePix Pro 5.0. Two slides were hybridized per strain with a dye-swap repeat per slide.

Hybridizations on *E. coli *virulence microarrays were performed as described previously [22]. Arrays were scanned with a ScanArray^® ^Lite fluorescent microarray analysis system (Canberra-Packard Canada, Montreal, Quebec) and acquisition and quantification of fluorescent spot intensities were performed using the ScanArray Express^® ^software version 2.1 (Perkin-Elmer, Foster City, CA, USA).

### Microarray data analysis

Data obtained from *E. coli *O157:H7 microarrays were normalized using the Ratio-based and Lowess methods in Acuity 3.1 (Axon instruments) before analysis. The normalized data for all strains were converted into log_2 _(Fluor 647/Fluor 546) in Acuity 3.1 and subsequently analyzed in Microsoft Excel. Control, blank, and test spots with a mean intensity below that of the mean of all negative controls were removed from the analysis. The arithmetic mean of the remaining spots across the four duplicates was calculated to construct the dataset. GACK (for Genomotyping Analysis by Charles Kim) [26], was used to generate a cut-off value determining the presence or absence of genes, and a dendrogam using the Euclidean distance metric with average linkage was created with tMEV v4.1 [27].

For the data obtained from *E. coli *virulence microarrays, the local background was subtracted from the recorded spot intensities. The median value of each set of triplicate spotted oligonucleotides was then compared to the median value of the negative control spots present on the array. Oligonucleotides with a signal-to-noise fluorescence ratio greater than 2.0 were considered as positive.

### Microarray data accession number

The microarray data have been deposited in NCBI's Gene Expression Omnibus (GEO accession number GSE17036) .

### PCR experiments

PCR experiments were performed for the nine O45 PEPEC strains and the REPEC strain E22 to determine the localization in their chromosome of the LEE and of the OI#122, as well as the integrity of the OI#122 and of the secondary type III secretion system gene cluster designated ETT2 (for *E. coli *type III secretion 2). All PCR experiments were performed as described in previous studies carried out on the LEE, OI#122 and ETT2 gene clusters (Additional file 1: Table S1 [21,23,28-31]).

PCR experiments were also performed for *nleA *and *nleC *genes. The pairs of primers used were nleA-F (ACCGCAATCCGAATTACCTC) – nleA-R (TCCATTGCGCGTATATCAGC) and ECs1812F (CTGTCCAACAGGGATAC) – ECs1812R (CCGCAATCCGAATTACC) for *nleA*, and nleC-F (AAGTGTAATACGCGCCGTCC) – nleC-R (ATCAGGACTCGCCTCATATC) and ECs0847F (CCCATTGCTCCTAATCG) – ECs0847R (CAGCGGAATACTCTGTG) for *nleC*. The conditions for amplifications were an initial denaturation of 95°C for 5 min, followed by 30 cycles of 95°C for 30 s; 55°C for 30 s; 72°C for 80 s and a final elongation of 72°C for 10 min.

## Results

### Characterization of O45 PEPEC strains using the *E. coli *virulence microarray

All O45 PEPEC strains and the REPEC strain E22 were characterized using the *E. coli *virulence microarray described previously, which includes probes targeting virulence genes generally found in AEEC but also virulence genes from the other *E. coli *virotypes [22]. All strains possessed their own specific virulence gene profile but were all classified as atypical EPEC (Additional file 2: Table S2 [32]). They all possessed the LEE genes and shared the same LEE profile: *eae(β) *– *espA *group I – *espB *group III – *tir *group I. In addition, each strain lacked the Shiga toxin 2 encoding genes *stx2A *and *stx2B*, as well as the bundle forming pili (BFP) encoding gene *bfpA *and the *E. coli *adherence factor (EAF) virulence plasmid marker *eaf*. Remarkably, all O45 PEPEC strains and REPEC strain E22, although *stx1A*-negative, gave a positive hybridization for the *stx1B *gene, which encodes the B subunit of EHEC Shiga-like toxin 1 and which is generally absent in EPEC strains. However, the presence of the *stx1B *gene was not confirmed in PCR experiments (data not shown).

The *E. coli *strains could be classified into two distinct groups according to their virulence gene pattern (Additional file 2: Table S2 [32]). Group I included the four PEPEC strains ECL1001, ECL2004, ECL2017, and ECL2033, and REPEC strain E22. Group II included the five other PEPEC strains ECL2019, ECL2020, ECL2027, ECL2076, and ECL2078. Results obtained with the *E. coli *virulence microarray identified 19 virulence genes that showed a non-random distribution between Group I and Group II strains (Additional file 2: Table S2 [32]). Genes *b1121 *(encoding a hypothetical protein YcfZ), *set *(encoding a probable enterotoxin, also named *ent*), *tspE4.C2 *(an anonymous fragment), *efa1 *(encoding the EHEC factor for adherence Efa1), and *paa *were present in all Group I strains, including REPEC strain E22, but absent from all Group II strains. The temperature sensitive hemagglutinin encoding gene *tsh*, the yersiniabactin-related genes *fyuA*, *irp1 *and *irp2*, as well as the PAI-associated gene *malX *were present in all O45 PEPEC strains from Group I but in neither REPEC strain E22 nor Group II strains. The heat-stable enterotoxin encoding gene *astA *was present in all O45 PEPEC Group I strains and in Group II strain ECL2020 but absent from all other Group II strains and REPEC strain E22.

The genes *aidaI *(encoding the Adhesin Involved in *E. coli *Diffuse Adherence), *chuA *(an iron related gene), *ECs1282 *(encoding a probable filamentous hemagglutinin-like protein), *rtx *(encoding a putative RTX family exoprotein), and *yjaA *(encoding a hypothetical protein) were present in all Group II strains but absent from Group I strains, including REPEC strain E22. The gene *fepC *(encoding a ferric enterobactin transport ATP-binding protein) was present in three Group II strains (ECL2019, ECL2078 and ECL2027) but absent from all other strains.

In addition, Group I and Group II strains possessed different variants of the *fliC *(encoding the flagellin major subunit) and *lpfA *genes. O45 PEPEC strains from Group I, including REPEC strain E22, possessed the *fliC *variant *flmA54*, whereas Group II strains possessed the *fliC *gene. Group I strains also possessed the *lpfA*_O113 _and *lpfA*_R141 _variants, whereas REPEC strain E22 only possessed the *lpfA*_R141 _variant, and Group II strains possessed the *lpfA1 *variant.

Previous phylogenetic analyses have shown that most *E. coli *strains belonged to the four main phylogenetic groups A, B1, B2, and D. Whereas extraintestinal *E. coli *strains belong mainly to groups B2 and D, most commensal and diarrheogenic strains belong to group A and group B1 [33]. Determination of the phylogenetic groups of the O45 PEPEC strains and REPEC strain E22 was based on the presence or absence of the two genes *chuA *and *yjaA*, and the DNA fragment *tspE4.C2*, as described by Clermont *et al*. [32]. All Group I strains including REPEC strain E22 were classified in phylogenetic group B1 and all Group II strains were classified in phylogenetic group B2.

### CGH-Genomotyping of PEPEC strains

**Figure 1:**
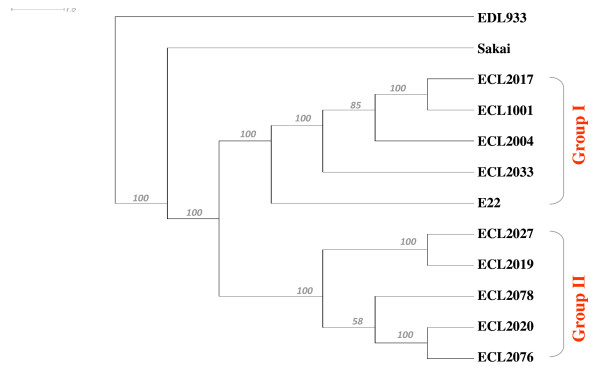
**CGH-based genomotyping of the O45 PEPEC strains**. The nine O45 PEPEC strains and the REPEC strain E22 were classified in two distinct groups by CGH-based genomotyping, in the same distribution as observed by the *E. coli *virulence microarray (Groups I and II). The O157:H7 strains Sakai and EDL933 were used as controls. The tree was constructed with tMEV v4.1 and viewed in SplitsTree 4.1 [27] by using the Euclidean distance, average linkage algorithm and 1,000 bootstrap replicates. Bootstrap confidence values are indicated at the nodes.

The CGH-based genomotyping analysis of the nine O45 PEPEC strains and the REPEC strain E22 led to their classification into two distinct groups in the same distribution as observed by the *E. coli *virulence microarray. A dendrogram based on the analysis of the CGH data for O45 PEPEC strains and REPEC strain E22, as well as for the two O157:H7 strains Sakai and EDL933 is presented in Figure 1. The distribution of GIs in O45 PEPEC strains and REPEC strain E22 was investigated by analysis of the CGH data. Since the microarray used for CGH was not an EPEC-specific microarray and was composed of oligonucleotide probes specific for genome sequences of O157:H7 EHEC and K12 strains, it was not possible to investigate all the GIs in O45 PEPEC strains. The divergences in GIs observed by CGH could thus indicate either the absence of particular genes or the presence of different variants of these genes. As shown in Table 2, 63 GIs (islands or loops) were found to be significantly different between Group I and Group II strains. Among these 63 GIs, 13 were KIs or K-loops and 50 were OIs or S-loops. Twenty GIs were present in Group I strains but absent in Group II strains, including OI#57 (S-loop#85), which contains the *paa *gene, and the two virulence related GIs, OI#71 (S-loop#108) [34] and OI#122 [35,36]. On the other hand, 33 GIs were absent in Group I strains but present in Group II strains, including OI#1 (S-loop#1), OI#47 (S-loop#71) and OI#154 (S-loop#253), which contain fimbriae related genes. KI#60 (K-loop#97), which was absent in all Group II strains, was present in the four O45 PEPEC strains from Group I but not in REPEC strain E22. Interestingly, for seven GIs, more than half of the ORFs in each island were present in all Group I strains whereas these GIs were absent in all Group II strains. Conversely, for two GIs, more than half of the ORFs in each island were present in Group II strains whereas these GIs were absent in Group I strains.

**Table 2:** Genomic islands comparison between two genotype groups of *E. coli *O45 strains.

K-Loop	K-island	O-island	S-loop	Group I^a^	Group II^a^	Notable function of this Island or Loop
		OI#1	S-loop#1	-	+	putative fimbrial chaperone and protein
		OI#2	S-loop#3	-	+	CcdA-like protein, CcdB-like protein
		OI#7	S-loop#14	+/-	-	VgrG protein
		OI#9	S-loop#17	-	+	putative transcriptional regulator, transport protein
		OI#11	S-loop#19	-	+	putative transcriptional regulator
		OI#14	S-loop#23	-	+	putative invertase, hypothetical protein
		OI#15	S-loop#24	+	-	Aida-I, adhesin-like protein
		OI#19	S-loop#30	-	+	hypothetical protein
		OI#20	S-loop#31	-	+	putative sensor histidine protein kinase
		OI#24	S-loop#37	-	+	hypothetical protein
		OI#25	S-loop#38	-	+	hypothetical protein
		OI#26	S-loop#39	-	+	hypothetical protein
		OI#28	S-loop#42	-	+	putative outer membrane transport protein
		OI#29	S-loop#43	-	+	adhesin/invasin-like protein
		OI#30	S-loop#44	+/-	-	putative Vgr protein
			S-loop#51	-	+	putative fimbrial-like protein
		OI#35	S-loop#52	-	+	putative transcription regulator
		OI#37	S-loop#57	-	+	hypothetical protein
		OI#41	S-loop#61	-	+	hypothetical protein
		OI#47	S-loop#71	-	+	hemagglutinin/hemolysin-related protein
		OI#50		+/-	-	putative antirepressor of prophage CP-933N
		OI#51	S-loop#78	+/-	-	putative single stranded DNA-binding protein
			S-loop#85	+	-	putative regulatory protein
		OI#52	S-loop#108	+	-	putative phage tail protein
		OI#52	S-loop#153	+	-	putative host specificity protein
		OI#57	S-loop#85	+	-	virulence factor Paa
		OI#65	S-loop#99	+	-	VgrE protein
		OI#70	S-loop#87	-	+	putative transcription regulatory protein
		OI#71	S-loop#108	+/-	-	BfpT-regulated chaperone-like protein
		OI#76	S-loop#119	+/-	-	putative transcriptional regulator
		OI#91		+	-	unknown function
		OI#106		-	+	putative polyferredoxin
		OI#115		-	+	TTSS or ETT2
		OI#120		+	-	hypothetical lipoprotein
		OI#122		+/-	-	putative adherence factor, putative enterotoxin
		OI#123		-	+/-	putative ABC-type iron-siderophore transport system
		OI#130		-	+	leader peptidase HopD
		OI#132		-	+	hypothetical protein
		OI#134	S-loop#220	-	+	putative ATP-dependent DNA helicase
		OI#136	S-loop#223	-	+	putative membrane protein
		OI#137	S-loop#224	-	+	hypothetical protein
		OI#140	S-loop#231	-	+	heme utilization/transport protein,
		OI#141	S-loop#232	-	+/-	putative fimbrial protein precursor
		OI#154	S-loop#253	-	+	putative type 1 fimbrial protein
		OI#155	S-loop#256	-	+	hypothetical protein
		OI#156	S-loop#257	+	-	hypothetical membrane protein
		OI#158	S-loop#264	-	+	hypothetical protein
		OI#159	S-loop#265	-	+	putative glycoprotein
		OI#161	S-loop#267	-	+	hypothetical membrane protein
		OI#162		+	-	RhsC protein in rhs element
K-loop#28	KI #18			+	-	polysaccharide metabolism; YaiP
K-loop#67	KI #40			+	-	hypothetical protein
K-loop#76	KI #44			+	-	hypothetical protein
K-loop#88	KI #52			+	-	homolog of virulence factor; *b1121*
K-loop#97	KI #60			+^b^	-	orf, hypothetical protein; YchG
K-loop#140	KI #84			-	+	orf, hypothetical protein; *b2071*
K-loop#164	KI #93			-	+	acetyl-CoA: acetoacetyl-CoA transferase alpha subunit
K-loop#178	KI #103			+	-	xanthosine permease; XapA, XapB
K-loop#203	KI #120			+	-	putative DEOR-type transcriptional regulator; YgbI
K-loop#218	KI #132			+	-	hypothetical protein
K-loop#220	KI #133			+	-	putative fimbrial-like protein; YgiL
K-loop#255	KI #154			+	-	putative lipase; YiaL
K-loop#267	KI #161			+	-	regulator protein for dgo operon; YidW

^a ^+/- indicates that more than 50% of the ORFs in this island or loop are present.

^b ^REPEC strain E22 is "-".

In addition to the 63 GIs found to be significantly different between Group I and Group II strains, 26 other GIs were conserved in both Groups (Table 3). Eight of these GIs were KIs or K-loops and 18 were OIs or S-loops.

**Table 3:** Conserved genomic islands identified by CGH in O45 PEPEC strains.

K-loop	K-island	O-island	S-loop	Number of ORFs	Notable function of this Island or Loop (if known)
		OI#17	S-loop #27	5	ribose transport related protein
			S-loop #45	1	
		OI #32		1	
		OI #61	S-loop #104	2	fimbrial protein
		OI #69	S-loop #89	2	
		OI #71	S-loop #109	1	chaperone protein
		OI #94		1	
		OI #103	S-loop #173	2	
		OI #108	S-loop #56	1	chaperone protein
		OI #110	S-loop #191	4	decarboxylase protein
			S-loop #192	8	
		OI#118		1	
		OI#119	S-loop #202	5	ABC transport protein
		OI#120	S-loop #203	1	
		OI#126	S-loop #211	2	sugar PTS protein
		OI#130	S-loop #215	1	bacterioferritin protein
		OI#145	S-loop #239	7	LPS biosynthesis protein
		OI#169	S-loop #278	2	
K-loop#90	KI #56			12	
K-loop#108	KI #66			20	
K-loop#115	KI #71			1	
K-loop#119	KI #73			1	
K-loop#126	KI #76			1	
K-loop#134	KI #80			1	
K-loop#232	KI #141			8	
K-loop#271	KI#163			8	

Finally, analysis of the *E. coli *O157:H7 microarray data indicated that the Shiga toxin encoding genes *stx1 *and *stx2 *could not be detected in any of the O45 PEPEC strains or in the REPEC strain E22, and that similarly, all strains were lacking both the *tccp *(ECs2715/Z3072) and *tccp2 *(ECs1126/Z1385) genes, which encode *E. coli *O157:H7 type III effector proteins that couple the intimin receptor Tir to the actin-cytoskeleton, and trigger actin polymerization [37-40].

### Analysis of LEE by the *E. coli *O157:H7 microarray

Thirty of the 41 genes on LEE (OI#148/S-loop#244) were found to be conserved among the O45 PEPEC strains, REPEC strain E22, and the two O157:H7 strains EDL933 and Sakai (Table 4). These included the effector-encoding genes *espA*, *espF *and *espG*, the regulator *ler*, and most of the genes of the type III secretion pathway such as *sepL*, *escD*, *cesT*, *escN*, *escV*, *sepD*, *escC*, *cesD*, *escU*, *escT*, *escS*, and *escR*. For the 11 remaining genes on LEE, no hybridization was observed in the O45 PEPEC strains and REPEC strain E22, possibly reflecting genetic divergences between these strains and the O157:H7 representative strains EDL933 and Sakai. These genes were the effector-encoding genes *espB*, *espD*, and *esp*H, intimin and the translocated intimin receptor-encoding genes *eae *and *tir*, the genes of the type III secretion pathway *sepQ*, *sepZ*, and *escJ*, and the genes *map*, *mpc *(for multiple point controller) and Z5117.

**Table 4:** Divergence in the LEE genes among O45 PEPEC strains and REPEC strain E22.

Divergent genes^a, b^	Group I strains^c^					Group II strains^c^					Function
			
	ECL1001	ECL2017	ECL2004	ECL2033	E22	ECL2019	ECL2078	ECL2027	ECL2020	ECL2076	
			
Z5105/ECs4554	-	+	-	-	-	-	-	-	-	-	EspB protein
Z5106/ECs4555	+	+	+	+	-	+	-	+	-	+	EspD protein
Z5110/ECs4559	-	-	-	-	-	-	-	-	-	-	Gamma intimin
Z5112/ECs4561	+	+	+	-	-	-	-	-	-	+	translocated intimin receptor Tir
Z5113/ECs4562	-	+	+	+	-	-	-	+	-	+	Map protein
Z5115/ECs4564	-	-	-	-	-	-	-	-	-	-	EspH protein
Z5116/ECs4565	-	+	+	-	+	+	-	+	-	+	type III secretion system SepQ protein
Z5117/ECs4566	-	+	+	+	-	+	-	+	-	+	hypothetical protein
Z5121/ECs4570	-	-	-	-	-	-	-	-	-	-	Mpc protein
Z5122/ECs4571	-	-	-	-	-	-	-	-	-	-	type III secretion system SepZ protein
Z5124/ECs4573	+	+	+	+	-	+	-	+	-	+	type III secretion system EscJ protein

^a ^Z, nomenclature for genes from O157:H7 strain EDL933; ECs, nomenclature for genes from O157:H7 strain Sakai.

^b ^the following genes were present in all O45 EPEC strains and REPEC strain E22: Z5100/ECs4550 (EspF protein), Z5102/ECs4551 (hypothetical protein), Z5103/ECs4552 (EscF protein), Z5104/ECs4553 (hypothetical protein), Z5107/ECs4556 (EspA protein), Z5108/ECs4557 (type III secretion system SepL protein), Z5109/ECs4558 (type III secretion system EscD protein), Z5111/ECs4560 (CesT protein), Z5114/ECs4563 (hypothetical protein), Z5118/ECs4567 (hypothetical protein), Z5119/ECs4568 (type III secretion system protein EscN), Z5120/ECs4569 (type III secretion system EscV protein), Z5123/ECs4572 (hypothetical protein), Z5125/ECs4574 (type III secretion system protein SepD), Z5126/ECs4575 (type III secretion system EscC protein), Z5127/ECs4576 (type III secretion system CesD protein), Z5128/ECs4577 (hypothetical protein), Z5129/ECs4578 (hypothetical protein), Z5131/ECs4579 (hypothetical protein), Z5132/ECs4580 (type III secretion system EscU protein), Z5133/ECs4581 (type III secretion system EscT protein), Z5134/ECs4582 (type III secretion system EscS protein), Z5135/ECs4583 (type III secretion system EscR protein), Z5136/ECs4584 (hypothetical protein), Z5137/ECs4585 (hypothetical protein), Z5138/ECs4586 (hypothetical protein), Z5139/ECs4587 (hypothetical protein), Z5140/ECs4588 (Ler protein), Z5142/ECs4590 (EspG protein), and Z5143/ECs4591 (hypothetical protein).

^c ^+, gene present; -, gene absent.

### Localization of LEE and OI#122

The LEE of AEEC strains is often inserted in the vicinity of the tRNA loci *selC *or *pheU*. Since it has been previously reported that the site of insertion of LEE in PEPEC strains could be either in *selC *or in *pheU *[23], the O45 PEPEC strains in our study and REPEC strain E22 were examined by PCR using primers specific for these two genes and for LEE extremities (Additional file 1: Table S1 [21,23,28-31]). The LEE was found to be inserted into the tRNA *pheU *locus in all examined strains. Remarkably, an amplicon of 500 bp longer than the expected size was also obtained with primers specific for LEE extremities and *selC *for strains ECL2033 and ECL2020 (Additional file 3: Table S3 [22]).

Similarly, the localization and integrity of OI#122 was determined by PCR using primers described previously (Additional file 1: Table S1 [21,23,28-31]). All Group I strains possessed this GI and were positive for the four genes tested; *efa1*, *ent*, *nleB*, and *nleE*, with the latter two encoding non-LEE virulence factors. OI#122 was found to be inserted into the tRNA *pheU *locus in strain ECL2033 and into the tRNA *pheV *locus in strains ECL1001 and ECL2004. The site of insertion of this GI was not determined for strain ECL2017 or for REPEC strain E22. All Group II strains lacked OI#122 (Additional file 3: Table S3 [22]).

### *nle *genes in O45 PEPEC strains

The *E. coli *O157:H7 microarray used in our CGH studies contains oligonucleotide probes specific for genes encoding non-LEE factors which have previously been associated with the pathogenicity of AEEC strains [34,41]. The *nleA *and *nleC *genes were present in all O45 PEPEC strains and REPEC strain E22 as determined by the O157:H7 microarray (Table 5). Nevertheless, PCR analysis using two different primer sets for each gene (Additional file 1: Table S1 [21,23,28-31]) revealed amplicons of various sizes in the different strains, showing that the *nleA *and *nleC *genes in Group I were different from those in Group II (data not shown).

**Table 5:** Distribution of *nle *genes among O45 PEPEC strains and REPEC strain E22.

			Group I strains					Group II strains				
				
Gene	ID^a^	Family	ECL1001	ECL2017	ECL2004	ECL2033	E22	ECL2019	ECL2078	ECL2027	ECL2020	ECL2076
*espY3*	Z0521 (ECs0472)	SopD-N; PRR	-	-	-	-	-	+	+	+	+	+
*espX2*	Z1019 (ECs0876)	PPR	-	-	-	-	-	+	+	+	+	+
*espR1*	Z2242 (ECs2073)	LRR	-	-	-	-	-	+	+	+	+	+
*espL3'*	Z5199 (ECs4642)	AR	-	-	-	-	-	+	+	+	+	+
*espL3'*	Z5200 (ECs4643)	AR	-	-	-	-	-	+	+	+	+	+
*espX7*	Z1822 (ECs1560)	PPR; LRR	+	+	+	+	+	-	-	-	-	-
*espK*	Z1829 (ECs1568)	LRR	+	+	+	+	+	-	-	-	-	-
*espL2*	Z4326 (ECs3855)	AR	+	+	+	+	+	-	-	-	-	-
*nleB1*	Z4328 (ECs3857)	NleB	+	+	+	+	+	-	-	-	-	-
*nleE*	Z4329 (ECs3858)	NleE	+	+	+	+	+	-	-	-	-	-
*nleB2-1*	Z0985 (ECs0846)	NleB	-	-	-	-	+	+	+	+	+	+
*nleG2-1'*	Z6025 (ECs1810)	NleG	+	+	+	+	-	-	-	-	-	-
*espO1-2*	ECs1821	OspE	+	+	+	+	-	-	-	-	-	-
*nleG*	Z6010 (ECs1824)	NleG	+	+	+	+	-	-	-	-	-	-
*nleG9'*	Z2560 (ECs1828)	NleG	+	+	+	+	-	-	-	-	-	-
*nleD*	Z0990 (ECs0850)	NleD	-	-	+	+	-	-	-	-	-	-
*nleF*	Z6020 (ECs1815)	NleF	+	+	+	+	-	+	+	+	+	+
*nleH*	Z0989 (ECs0848)	NleH	+	+	+	+	+	+	+	+	+	+
*nleA*^b^	Z6024 (ECs1812)	NleA	+	+	+	+	+	+	+	+	+	+
*nleC*^b^	Z0986 (ECs0847)	NleC	+	+	+	+	+	+	+	+	+	+

^a ^Z, nomenclature for O157:H7 strain EDL933; ECs, nomenclature for O157:H7 strain Sakai.

^b ^PCRs using two different primer sets for each gene revealed that both *nleA *and *nleC *in Group I were different from those in Group II.

Sixteen other *nle *genes showed non-random distributions between Group I and Group II strains (Table 5). The five genes *espY3*, *espX2*, *espR1*, *espL3' *(Z5199/ECs4642), and *espL3' *(Z5200/ECs4643) were absent in all Group I strains but present in Group II strains. On the other hand, the five genes *espX7*, *espK*, *espL2*, *nleB1*, and *nleE *were present in all Group I strains but absent in Group II strains. The four genes *nleG2-1'*, *espO1-2*, *nleG*, and *nleG9' *were present in all Group I strains with the exception of REPEC strain E22, and absent in Group II strains. The gene *nleB2-1 *was present in all Group II strains and also in REPEC strain E22 but absent in the other Group I strains. The gene *nleD *was present in only two Group I strains, ECL2004 and ECL2033, and absent in all the other strains, including REPEC strain E22.

Two additional *nle *genes, *nleF *and *nleH*, were present in all O45 PEPEC strains. *nleH*, but not *nleF*, was also present in REPEC strain E22 (Table 5).

### Distribution of ETT2 genes

OI#115, initially described in *E. coli *of serotype O157:H7 and present in other EPEC and EHEC strains from animals and humans, contains the secondary type III secretion system gene cluster ETT2 [31,42,43]. CGH data analysis revealed that all Group II strains had the entire ETT2 locus comprising 36 genes, with the exception of strain ECL2019, which lacked most of this GI (Additional file 4: Table S4 [16]).

In contrast, Group I strains possessed only a partially intact locus and the occurrence of the ETT2 genes was highly variable. Among the 36 ETT2 genes, strain ECL2033 possessed only 21, strain ECL2004 possessed 20, strain ECL1001 possessed 17, and strain ECL2017 possessed 15. Finally, REPEC strain E22 possessed 21 genes of this GI. These results were confirmed by PCR as described previously [31], with primers specific for different regions of the ETT2 gene cluster (Additional file 1: Table S1 [21,23,28-31]).

### Genes required for intestinal colonization in the bovine

Previous studies have identified several genes required for EHEC intestinal colonization of the bovine [44,45]. Microarray analysis in our study revealed that 13 genes associated with colonization of either *E. coli *O157:H7 or *E. coli *O26:H- in the bovine were associated with either Group I or Group II strains (Additional file 5: Table S5). Seven genes were present in Group I but not in Group II strains, with the exception of REPEC strain E22 which did not possess the gene Z6010 (ECs1824). In contrast, six other genes were present in Group II but not in Group I strains, with the exception of REPEC strain E22 which possessed the gene Z1526 (ECs1270). These results were confirmed by PCR using primers designed for each gene (data not shown).

## Discussion

In this study, we investigated the genetic relationships among PEPEC strains of serogroup O45 and catalogued genomic alterations unique to these strains by using both a virulence gene-specific microarray and a whole genome microarray. The 045 PEPEC strains in this study have been previously characterized for their capacity to induce A/E lesions in both explants and challenged pigs, and were grouped according to the severity of the A/E manifestation they produced [13]. Based on their virulence gene content as determined by the *E. coli *virulence microarray, O45 PEPEC strains and REPEC strain E22 displayed significant differences from typical EPEC and could be regarded as atypical EPEC, that are defined as LEE-positive *E. coli *lacking *stx*_1 _and *stx*_2 _genes, as well as the EAF virulence plasmid which encodes the EPEC adhesin BFP [46,47]. In addition, all O45 PEPEC strains and REPEC strain E22 unexpectedly hybridized with the *stxB*_1 _probe of the *E. coli *virulence microarray, as was also observed for some atypical EPEC strains isolated from children with diarrhea in a recent study in Norway [35,48]. Due to the absence of hybridization with the corresponding *stxA*_1 _probe and the negative PCR results obtained with *stxB*_1 _sequence specific primers [35,48], we therefore concluded that the gene sequences detected by the *stxB*_1 _hybridization probe did not represent a complete *stxB *gene but rather a possible truncated form of this gene.

As observed for other atypical EPEC strains, O45 PEPEC strains and REPEC strain E22 also displayed a relatively high heterogeneity in their virulence gene profiles [35,49]. Based on their virulence gene content, they could be divided into two distinct groups, Groups I and II. It has been argued that atypical EPEC strains could have arisen from *E. coli *strains of different pathotypes which acquired the LEE by horizontal gene transfer or from certain typical EPEC strains that have lost the EAF plasmid [49]. Trabulsi *et al*. have also observed that some atypical EPEC strains are genetically closer to EHEC strains of serotype O157:H7 than to typical EPEC [50]. Several virulence genes showed a non-random distribution between Group I and Group II strains. Group I strains thus possessed several virulence-related genes which were absent in Group II strains. Group I-specific genes included *paa *(which contributes to A/E lesion formation in PEPEC strains [10]) and OI#122 genes *efa1 *(which plays an important role in intestinal colonization by EHEC strains in cattle [51]) and *set *(which encodes a putative enterotoxin highly similar to the enterotoxin ShET2 of *Shigella flexneri*). Genes associated with other pathotypes were also found. The gene *tsh*, encoding a hemagglutinin which may be a virulence factor of avian extraintestinal *E. coli *[52], the pathogenicity island marker *malX*, related to virulence in extraintestinal *E. coli *[53] and the yersiniabactin-related genes *fyuA*, *irp1 *and *irp2*, implicated in the ferric uptake system, were also Group I-specific. In contrast, Group II strains possessed only a few additional virulence-related genes when compared with Group I strains. These included *aidaI*, which encodes a protein involved in the adherence of EPEC [54], and iron uptake-related genes *chuA *and *fepC*. Finally, Group I and Group II strains also possessed different variants of the long polar fimbriae encoding gene *lpfA*. A recent study has shown that the *lpfA*_O113 _variant, found in Group I strains in our study, was found significantly more frequently in atypical EPEC strains associated with cases of diarrhea than in strains isolated from healthy individuals [35].

It is interesting to note that analysis by CGH using a whole genome *E. coli *microarray, representing two lineage I, human outbreak-related *E. coli *O157:H7 strains and one non-pathogenic *E. coli *K12 strain, resulted in the distribution of the O45 PEPEC strains into the same two groups (Groups I and II), observed for the *E. coli *virulence microarray. This genetic-based grouping, principally reflecting the virulence gene content of the strains, was also compatible with the grouping based on their A/E activity [13]. The O45 PEPEC strains of Group I all induced severe A/E lesions whereas those of Group II induced less severe or no A/E lesions in both pig ileal explants and challenged pigs. REPEC strain E22 was placed into Group I but was genetically distant from the four O45 PEPEC strains belonging to this group. These strains showed a relatively high level of heterogeneity in their virulence gene profiles.

In addition to the variations in particular virulence genes, significant variations in GIs were also observed between Group I and II strains. We observed that several virulence-related OIs were present only in Group I strains. These included OI#57 (S-loop#85), which contains the *paa *gene; OI#71 (S-loop#108), which contains the non-LEE encoded factor gene *nleA*, previously shown to be associated with the pathogenicity of AEEC strains [34]; and OI#122 which contains the two non-LEE encoded factor genes *nleB *and *nleE *and virulence genes *efa1 *and *set*. Interestingly, these OIs have also been shown to be more prevalent in STEC strains associated with outbreaks and severe disease [35,36]. On the other hand, certain OIs were only present in Group II strains. These include OI#1 (S-loop#1), containing genes encoding putative fimbrial chaperone proteins; OI#47 (S-loop#71), containing a fimbrial operon and genes encoding several additional putative virulence factors in *E. coli *of serogroup O157 [55]; and OI#154 (S-loop#253), containing genes encoding putative type 1 fimbrial proteins. Finally, OI#115 was highly divergent between Group I and Group II strains. This OI contains the secondary type III secretion system gene cluster ETT2, which resembles the SPI-1-encoded type III secretion system from *Salmonella enterica *and has been previously characterized in O157:H7 *E. coli *strains [16,17]. It has been recently shown that ETT2 influences the secretion of proteins encoded by the LEE and also modulates adhesion to human intestinal cells [56]. Most Group II strains (4/5) possessed the entire ETT2. However, Group II strain ECL2019 lacked most of the entire cluster (only four genes were found to be present) and was one of the two strains which did not induce A/E lesions in explants or in challenged pigs. Group I strains, including REPEC strain E22, have only a partial ETT2 gene cluster, possessing from 15 to 21 genes of the 36 in the intact cluster. The substantial variations observed for this cluster are consistent with the findings of previous studies, that while the ETT2 gene cluster was present in most of the *E. coli *strains tested, it contained numerous inactivating mutations [31,42,43].

In contrast to the heterogeneity of their virulence gene content and GI distribution, O45 PEPEC strains and REPEC strain E22 showed a high level of homogeneity in their LEE sequences and site of insertion. In all strains, the LEE was inserted into the tRNA *pheU *gene and no significant divergence between Group I and Group II strains was observed for the LEE genes. In addition, all O45 PEPEC strains and REPEC strain E22 shared the same profile for the intimin encoding gene *eae*, its translocated receptor *tir *and the effector encoding genes *espA *and *espB*, as shown by the *E. coli *virulence microarray. All strains possessed the beta variant of the intimin encoding gene, being the most widespread among the intestinal EPEC strains of different animal species [57,58]. However, Group I and Group II strains from our study all belonged to the phylogenetic groups B1 and B2 whereas Ishii *et al*. have shown that most EPEC strains possessing the intimin subtype beta belong to phylogenetic groups A and B1 [59].

Finally, we have also observed numerous disparities in the distribution of non-LEE encoded genes. Several *nle *genes were only present in Group I strains whereas others were only found in Group II strains. In addition, Group I and Group II strains possessed two different variants of the genes *nleA *and *nleC*. This variation in the distribution of *nle *genes may influence the pathogenicity of the strains and the type of A/E lesions they produce, since many studies have shown the importance of non-LEE encoded factors in the A/E phenotype [34,41,60,61].

## Conclusion

We have genetically characterized a collection of O45 PEPEC strains using *E. coli *O157-*E. coli *K12 whole genome and virulence gene-specific *E. coli *microarrays. We have shown that the strains, although showing some heterogeneity, could be classified into two groups, based on their virulence gene and GI content. These differences in their virulence gene content may influence the pathogenicity of O45 PEPEC strains, and explain why Group I O45 PEPEC strains induced more severe A/E lesions in explants and challenged pigs than Group II strains [13,14].

## Authors' contributions

GB and YZ both contributed equally to the manuscript: they were involved in the conception and design of the study, in the analysis and interpretation of the microarray data, and in drafting and revising the manuscript. PG, JW, and CL were involved in the acquisition of the microarray data and in revising the manuscript. JMF was involved in revising the manuscript critically for important intellectual content. VPJG and JH were involved in the conception and design of the study, and revising the manuscript critically for important intellectual content. All authors read and approved the final manuscript.

## Supplementary Material

Additional file 1 **Table S1**. Primers and *E. coli *control strains used for PCR experiments. Click here for file

Additional file 2 **Table S2**. Presence of virulence genes in O45 PEPEC strains and REPEC strain E22 as determined by *E. coli *virulence microarray. Click here for file

Additional file 3 **Table S3**. Localization of the LEE and OI#122 in O45 PEPEC strains and REPEC strain E22. Click here for file

Additional file 4 **Table S4**. Distribution of ETT2 genes in O45 PEPEC strains and REPEC strain E22. Click here for file

Additional file 5 **Table S5**. Divergence of genes related to intestinal colonization in bovine and calves as determined by CGH. Click here for file
