# Supplementary material for: Two distinct groups of porcine enteropathogenic Escherichia coli strains of serogroup O45 are revealed by comparative genomic hybridization and virulence gene microarray
Source: BMC Genomics. 2009 Aug 26;10:402. doi: 10.1186/1471-2164-10-402 (PMC2749873; doi:10.1186/1471-2164-10-402)
Supplement: Additional file 1 — Table S1. Primers and E. coli control strains used for PCR experiments. [file 1471-2164-10-402-S1.pdf]

Table S1. Primers and *E. coli* control strains used for PCR experiments.

Primers	Target	Control strains	Reference[a]
Location of the LEE			
K260 / K261	*selC* integrity	EDL933	McDaniel *et al.* [28]
PheU-fl-1 / PheU-fl-2	*pheU* integrity	RDEC-1	Bielaszewska *et al.* [29]
K295 / K296	LEE inserted in *selC*, left junction	EDL933	An *et al.* [23]
K260 / K255	LEE inserted in *selC*, right junction	EDL933	McDaniel *et al.* [28]
K913 / K916	LEE inserted in *pheU*, left junction	RDEC-1	Sperandio *et al.* [21]
K913 / K917	LEE inserted in *pheU*, right junction	RDEC-1	Sperandio *et al.* [21]
Location and integrity of OI#122			
PheU-fl-1 / PheU-fl-2	*pheU* integrity	RDEC-1	Bielaszewska *et al.* [29]
PheV-F/ PheV-R	*pheV* integrity	MG1655	Bertin *et al.* [30]
PheU-r / P4-int	OI#122 next to *pheU*, right junction	CFT073	Bielaszewska *et al.* [29]
PheV-F / P4-int	OI#122 next to *pheV*, right junction	EDL 933	Bielaszewska *et al.* [29]
ent-f / ent-r	Presence of the *ent* gene	EDL933	Bielaszewska *et al.* [29]
nleB-f/ nleB-r	Presence of the *nleB* gene	EDL933	Bielaszewska *et al.* [29]
nleE-f/ nleE-r	Presence of the *nleE* gene	EDL933	Bielaszewska *et al.* [29]
Integrity of the ETT2 gene cluster			
prgEC-C1 / prgEC-C2	Presence of the *prgEC* operon	EDL933	Hartleib *et al.* [31]
invH-H1 / invH-H2	Presence of the *invH* gene	EDL933	Hartleib *et al.* [31]
spaS-1 / spaS-2	Presence of the *spaS* gene	EDL933	Hartleib *et al.* [31]
invEG-1 / invEG-2	Presence of the *invEG* operon	EDL933	Hartleib *et al.* [31]
orf-3737-F / orf-3737-R	Presence of the orf3737	EDL933	Hartleib *et al.* [31]
rmbA-F / rmbA-R	Presence of the *rmbA* gene	EDL933	Hartleib *et al.* [31]
Presence of nle genes			
nleA-F / nleA-R	Presence of the *nle*A gene	EDL933	This study[b]
ECs1812F / ECs1812R	Presence of the *nle*A gene	EDL933	This study[b]
nleC-F / nleC-R	Presence of the *nle*C gene	EDL933	This study[b]
ECs0847F / ECs0847R	Presence of the *nle*C gene	EDL933	This study[b]

[a] Studies in which PCR amplification conditions and primers were described.
[b] See materials and methods section.
