# Supplementary material for: Two distinct groups of porcine enteropathogenic Escherichia coli strains of serogroup O45 are revealed by comparative genomic hybridization and virulence gene microarray
Source: BMC Genomics. 2009 Aug 26;10:402. doi: 10.1186/1471-2164-10-402 (PMC2749873; doi:10.1186/1471-2164-10-402)
Supplement: Additional file 2 — Table S2. Presence of virulence genes in O45 PEPEC strains and REPEC strain E22 as determined by E. coli virulence microarray. [file 1471-2164-10-402-S2.pdf]

Table S2. Presence of virulence genes in O45 PEPEC strains and REPEC strain E22 as determined by *E. coli* virulence microarray.

Genes and functions[c]	Group I strains[a]					Group II strains[b]				
	ECL1001	ECL2017	ECL2004	ECL2033	E22	ECL2019	ECL2078	ECL2027	ECL2020	ECL2076
**adhesins**										
afr2G, fimbrial major subunit of pili AF/R2 (Adhesive factor/rabbit 2)	-	-	-	-	+	-	-	-	-	-
aida1, Adhesin Involved in Diffuse Adherence	-	-	-	-	-	+	+	+	+	+
csgE, assembly/transport component in curli production	+	+	+	+	+	+	+	+	+	+
fimA, major fimbrial subunit of type 1 fimbriae	+	+	+	+	+	+	+	+	-	+
fimH, adhesin of type 1 fimbriae	+	+	+	+	+	+	+	+	+	+
iha, adhesin Iha (IrgA homologue adhesin)	+	+	-	-	-	-	-	-	-	-
lpfA, major fimbrial subunit of fimbriae LPF (long polar fimbriae), variant *lpfA*$_{R141}$	+	+	+	+	+	-	-	-	-	-
lpfA(O113), major fimbrial subunit of fimbriae LPF (long polar fimbriae), variant *lpfA*$_{O113}$	+	+	+	+	-	-	-	-	-	-
lpfA(O157), major fimbrial subunit of fimbriae LPF (long polar fimbriae), variant *lpfA1*	-	-	-	-	-	+	+	+	+	+
**capsular and somatic antigens**										
wzy(O103), O antigen polymerase, O103 antigen	-	-	-	-	+	-	-	-	-	-
**colicins and microcins**										
ce1a, structural gene for colicin E1	+	+	-	-	-	-	-	-	-	-
**haemolysins and hemagglutins**										
hlyA, structural subunit of chromosomal hemolysin	-	-	-	-	-	-	-	+	+	-
hlyE, silent hemolysin, haemolytic phenotype when overexpressed, other name: sheA	+	+	+	+	+	+	+	+	+	+
tsh, temperature-sensitive hemagglutinin of avian *E. coli*, autotransporter	+	+	+	+	-	-	-	-	-	-
**iron acquisition or transport systems**										
chuA, outer membrane receptor protein, heme utilization/transport protein	-	-	-	-	-	+	+	+	+	+
fepC, ferric enterobactin transport ATP-binding protein	-	-	-	-	-	+	+	+	-	-
fyuA, pesticin/yersiniabactin receptor protein	+	+	+	+	-	-	-	-	-	-
irp1, Irp1: yersiniabactin biosynthetic protein	+	+	+	+	-	-	-	-	-	-
irp2, Irp2: yersiniabactin biosynthetic protein	+	+	+	+	-	-	-	-	-	-
**locus of enterocyte effacement (LEE)**										
eae, intimin (attaching and effacing lesions)	+	+	+	+	+	+	+	+	+	+
eae(beta), intimin, beta variant	+	+	+	+	+	+	+	+	+	+
espA-1, EspA protein secreted by the type III secretion system of the LEE, group I	+	+	+	+	+	+	+	+	+	+
espB-3, EspB protein secreted by type III secretion system of the LEE, group III	+	+	+	+	+	+	+	+	+	+
tir-1, translocated intimin recepetor, group I	+	+	+	+	+	+	+	+	+	+
**toxins**										
astA, enteroaggregative *E. coli* heat-stable enterotoxin 1, other name: eastI	+	+	+	+	-	-	-	-	+	-
stx1B, shiga-like toxin I, subunit B	+	+	+	+	+	+	+	+	+	+

[a] All Group I strains lacked the *chuA* gene and possessed the *tspE4.C2* fragment. As described by Clermont *et al.*, they were classified into phylogenetic group B1 [32].

[b] All Group II strains possessed the *chuA* and *yjaA* genes. As described by Clermont *et al.*, they were classified into phylogenetic group B2 [32].

[c] All strains possessed the genes *lacY-Ec* (*E. coli* lactose permease), *lacZ* (*E. coli* beta-galactosidase), *tnaA* (*E. coli* tryptophanase), *uidA* (*E. coli* beta-glucuronidase) and *gad* (glutamate decarboxylase A) which are the positive controls of the microarray.

Table S2 (continued). Presence of virulence genes in O45 PEPEC strains and REPEC strain E22 as determined by *E. coli* virulence microarray.

Genes and functions[c]	Group I strains[a]					Group II strains[b]				
	ECL1001	ECL2017	ECL2004	ECL2033	E22	ECL2019	ECL2078	ECL2027	ECL2020	ECL2076
newly recognized or putative virulence factors										
artJ, L-arginine periplasmic binding protein, supposed to be involved in virulence	+	+	+	+	+	+	+	+	+	+
b1121, Hypothetical protein YcfZ, homologous to virulence factor	+	+	+	+	+	-	-	-	-	-
ECs1282, probable filamentous hemagglutinin-like protein	-	-	-	-	-	+	+	+	+	+
mviM, putative virulence factor	+	+	+	+	+	+	+	+	+	+
mviN, putative virulence factor	+	+	+	+	+	+	+	+	+	+
rtx, putative RTX family exoprotein	-	-	-	-	-	+	+	+	+	+
set, probable enterotoxin, similar to ShET2 enterotoxin (*S. flexneri*), other name: ent	+	+	+	+	+	-	-	-	-	-
tspE4.C2, anonymous fragment (putative lipase)	+	+	+	+	+	-	-	-	-	-
yjaA, Hypothetical protein	-	-	-	-	-	+	+	+	+	+
various functions										
ccdB, cytotoxic protein, F-plasmid-encoded toxin	-	-	+	-	-	-	-	-	-	-
cif, *E. coli* cell cycle inhibiting factor	+	+	+	+	+	+	+	+	+	+
efa1, EHEC factor for adherence (efa1), identical to *lifA* (lymphocyte activation inhibitor)	+	+	+	+	+	-	-	-	-	-
fliC, *E. coli* flagellin, major subunit	-	-	-	-	-	+	+	+	+	+
flmA54, *E. coli* flagellin, major subunit, *fliC* variant	+	+	+	+	+	-	-	-	-	-
ibeB, invasion gene locus (penetration of brain microvascular endothelial cells), putative resistance protein, putative outer membrane lipoprotein of copper ion antiporter	+	+	+	+	+	+	+	+	+	+
iss, gene for increased serum survival (similar to Bacteriophage lambda Bor)	+	+	+	+	-	+	+	+	-	+
malX, maltose and glucose-specific IIABC component, pathogenicity island associated	+	+	+	+	-	-	-	-	-	-
ompA, outer membrane protein (OMPA or OMPII)	+	+	+	+	+	+	+	+	+	+
ompT, outer membrane protein 3b, other name: protease VII	+	+	+	+	+	+	+	+	+	+
paa, porcine attaching and effacing associated protein	+	+	+	+	+	-	-	-	-	-
nleA, non-LEE encoded effector A (type III secreted effector), *espI*-like	+	-	+	+	-	+	-	-	-	+
nleA(EHEC), non-LEE encoded effector A (type III secreted effector) of EHEC, *espI*-like	+	+	+	+	-	-	-	-	-	-
nleA(EPEC), non-LEE encoded effector A (type III secreted effector) of EPEC, *espI*-like	-	-	-	-	+	+	+	+	+	+
traT, complement resistance protein	+	+	+	-	-	+	+	+	+	+

[a] All Group I strains lacked the *chuA* gene and possessed the *tspE4.C2* fragment. As described by Clermont *et al.*, they were classified into phylogenetic group B1 [32].

[b] All Group II strains possessed the *chuA* and *yjaA* genes. As described by Clermont *et al.*, they were classified into phylogenetic group B2 [32].

[c] All strains possessed the genes *lacY-Ec* (*E. coli* lactose permease), *lacZ* (*E. coli* beta-galactosidase), *tnaA* (*E. coli* tryptophanase), *uidA* (*E. coli* beta-glucuronidase) and *gad* (glutamate decarboxylase A) which are the positive controls of the microarray.
