# Supplementary material for: Two distinct groups of porcine enteropathogenic Escherichia coli strains of serogroup O45 are revealed by comparative genomic hybridization and virulence gene microarray
Source: BMC Genomics. 2009 Aug 26;10:402. doi: 10.1186/1471-2164-10-402 (PMC2749873; doi:10.1186/1471-2164-10-402)
Supplement: Additional file 3 — Table S3. Localization of the LEE and OI#122 in O45 PEPEC strains and REPEC strain E22. [file 1471-2164-10-402-S3.pdf]

Table S3. Localization of the LEE and OI#122 in O45 PEPEC strains and REPEC strain E22.

Strains	LEE insertion site	Presence of OI#122	Contents of OI#122	Localisation of OI#122[c]
Group I strains				
ECL1001	*pheU*	Yes	*efa1*[b], *ent*, *nleB*, *nleE*	*pheV*
ECL2017	*pheU*	Yes	*efa1*[b], *ent*, *nleB*, *nleE*	Unknown
ECL2004	*pheU*	Yes	*efa1*[b], *ent*, *nleB*, *nleE*	*pheV*
ECL2033	*pheU* or *selC*[a]	Yes	*efa1*[b], *ent*, *nleB*, *nleE*	*pheU*
E22	*pheU*	Yes	*efa1*[b], *ent*, *nleB*, *nleE*	unknown
Group II strains				
ECL2019	*pheU*	No	None	NA
ECL2078	*pheU*	No	None	NA
ECL2027	*pheU*	No	None	NA
ECL2020	*pheU* or *selC*[a]	No	None	NA
ECL2076	*pheU*	No	None	NA

[a] One amplicon of expected size with primers specific to LEE and *pheU* and one amplicon 500bp greater in size with primers specific to LEE and *selC*.

[b] This gene was not tested by PCR but with the *E. coli* virulence microarray [22].

[c] NA, not applicable.
