# Supplementary material for: Two distinct groups of porcine enteropathogenic Escherichia coli strains of serogroup O45 are revealed by comparative genomic hybridization and virulence gene microarray
Source: BMC Genomics. 2009 Aug 26;10:402. doi: 10.1186/1471-2164-10-402 (PMC2749873; doi:10.1186/1471-2164-10-402)
Supplement: Additional file 4 — Table S4. Distribution of ETT2 genes in O45 PEPEC strains and REPEC strain E22. [file 1471-2164-10-402-S4.pdf]

Table S4. Distribution of ETT2 genes in O45 PEPEC strains and REPEC strain E22.

	Group I strains					Group II strains					
Gene[a]	ECL1001	ECL2017	ECL2004	ECL2033	E22	ECL2019	ECL2078	ECL2027	ECL2020	ECL2076	Function
Z4166 (ECs3703)	+	+	+	+	+	-	+	+	+	+	orf, hypothetical protein; YqeH
Z4167 (ECs3704)	+	+	+	+	+	-	+	+	+	+	putative sensory transducer; YqeI
Z4168 (ECs3705)	+	+	+	+	+	-	+	+	+	+	orf, hypothetical protein; YqeJ
Z4169 (ECs3706)	+	+	+	+	-	-	+	+	+	+	orf, hypothetical protein; YqeK
Z4170[b]	-	+	+	+	-	-	+	+	+	+	orf; Unknown function
Z4171 (ECs3707)	+	+	+	+	-	+	+	+	+	+	orf, hypothetical protein; YgeF
Z4172 (ECs3708)	+	+	+	+	-	-	+	+	+	+	orf, hypothetical protein; YgeG
Z4173 (ECs3709)	+	+	+	+	-	-	+	+	+	+	putative invasion protein; YgeH
Z4174 (ECs3710)	+	+	+	+	-	-	+	+	+	+	orf, hypothetical protein; b2853
Z4175 (ECs3711)	+	+	+	+	+	-	+	+	+	+	orf, hypothetical protein; b2854
Z4176 (ECs3712)	+	+	+	+	+	-	+	+	+	+	putative 2-component transcriptional regulator; YgeK
Z4177[b]	+	+	+	+	+	-	+	+	+	+	orf; Unknown function
Z4178 (ECs3713)	+	+	+	+	+	+	+	+	+	+	orf, hypothetical protein; b2857
Z4179 (ECs3714)	+	+	+	+	+	+	+	+	+	+	orf, hypothetical protein; b2858
Z4180 (ECs3716)	-	-	-	+	+	-	+	+	+	+	type III secretion system lipoprotein precursor; Eprk
Z4181 (ECs3717)	-	-	-	+	+	-	+	+	+	+	type III secretion protein; EprJ
Z4182 (ECs3719)	-	-	+	+	+	-	+	+	+	+	type III secretion protein; EprH
Z4183[b]	-	-	-	+	+	-	+	+	+	+	orf; Unknown function
Z4184 (ECs3720)	-	-	+	+	+	-	+	+	+	+	putative transcriptional regulator
Z4185 (ECs3721)	-	-	-	+	+	-	+	+	+	+	type III secretion protein; EprS
Z4186 (ECs3722)	-	-	-	-	+	-	+	+	+	+	type III secretion protein; EpaR2
Z4187 (ECs3723)	-	-	-	-	-	-	+	+	+	+	type III secretion protein; EpaR1
Z4188 (ECs3724)	-	-	-	-	+	-	+	+	+	+	type III secretion protein; EpaQ
Z4189 (ECs3725)	-	-	-	-	+	-	+	+	+	+	type III secretion protein; EpaP
Z4190 (ECs3726)	-	-	+	-	+	-	+	+	+	+	type III secretion protein; EpaO
Z4191 (ECs3727)	-	-	-	-	+	-	+	+	+	+	type III secretion protein; EivJ
Z4192 (ECs3728)	-	-	-	-	-	-	+	+	+	+	hypothetical protein
Z4193 (ECs3729)	+	-	+	-	-	-	+	+	+	+	type III secretion protein; EivI
Z4194 (ECs3730)	-	-	-	-	-	-	+	+	+	+	type III secretion protein ATP synthetase; EivC
Z4195 (ECs3731)	-	-	-	-	-	-	+	+	+	+	type III secretion protein; EivA
Z4196 (ECs3732)	-	-	-	+	-	-	+	+	+	+	type III secretion protein; EivE
Z4197 (ECs3733)	-	-	+	-	-	-	+	+	+	+	type III secretion protein; EivG
Z4198 (ECs3734)	+	-	-	-	-	-	+	+	+	+	type III secretion protein; EivF
Z4199 (ECs3735)	+	-	-	-	-	-	+	+	+	+	hypothetical protein
Z4200 (ECs3736)	-	-	-	-	+	-	+	+	+	+	hypothetical protein
Z4201 (ECs3737)	+	+	+	-	+	+	+	+	+	+	orf, hypothetical protein; b2863

[a] Z, nomenclature for O157:H7 strain EDL933; ECs, nomenclature for O157:H7 strain Sakai.

[b] The sequence corresponding to this probe is present in O157:H7 strain Sakai but no ORF is described in its genome's annotations [16].
