# Supplementary material for: Two distinct groups of porcine enteropathogenic Escherichia coli strains of serogroup O45 are revealed by comparative genomic hybridization and virulence gene microarray
Source: BMC Genomics. 2009 Aug 26;10:402. doi: 10.1186/1471-2164-10-402 (PMC2749873; doi:10.1186/1471-2164-10-402)
Supplement: Additional file 5 — Table S5. Divergence of genes related to intestinal colonization in bovine and calves as determined by CGH. [file 1471-2164-10-402-S5.pdf]

Table S5. Divergence of genes related to intestinal colonization in bovine and calves as determined by CGH.

Genes	Group I strains[a, b]					Group II strains[a, b]					Functions
	ECL1001	ECL2017	ECL2004	ECL2033	E22	ECL2019	ECL2078	ECL2027	ECL2020	ECL2076	
Z0609 (ECs0541)	-	-	-	-	-	+	+	+	+	+	Putative role in membrane transport of large molecules
Z5444 (ECs4827)	-	-	-	-	-	+	+	+	+	+	Putative glycoporin
Z3948 (ECs3515)	-	-	-	-	-	+	+	+	+	+	Putative ATP-binding component of a transport system, *ypjA*
Z3950 (ECs3517)	-	-	-	-	-	+	+	+	+	+	ORF, unknown function, but upstream *ypjA*
Z3496 (ECs3123)	-	-	-	-	-	+	+	+	+	+	ORF, unknown function
Z1526 (ECs1270)	-	-	-	-	+	+	+	+	+	+	Putative outer-membrane protein, *ycdS*
Z0275 (ECs0245)	+	+	+	+	+	-	-	-	-	-	Putative H repeat-associated protein
Z1930 (ECs1662)	+	+	+	+	+	-	-	-	-	-	Putative protease encoded within prophage CP-933X
Z2144 (ECs2162)	+	+	+	+	+	-	-	-	-	-	Putative tail component of prophage CP-933O
Z2318 (ECs2011)	+	+	+	+	+	-	-	-	-	-	Putative phosphatidate cytidyltransferase
Z2368	+	+	+	+	+	-	-	-	-	-	Unknown protein encoded within prophage CP-933R
Z4326 (ECs3855)	+	+	+	+	+	-	-	-	-	-	Putative enterotoxin
Z6010 (ECs1824)	+	+	+	+	-	-	-	-	-	-	ORF, unknown function

[a] +, presence of the gene; -, absence of the gene.
[b] all microarray results have been confirmed by PCR.
